# Opportunities and perspectives for developing orexin receptor antagonists

**DOI:** 10.3389/fnins.2014.00158

**Published:** 2014-06-12

**Authors:** Michel A. Steiner, Christopher J. Winrow

**Affiliations:** ^1^Actelion Pharmaceuticals Ltd., Department of Central Nervous System (CNS) PharmacologyAllschwil, Switzerland; ^2^Merck Research Laboratories, Department of NeuroscienceWest Point, PA, USA

**Keywords:** orexin, hypocretin, neuropeptide, orexin receptor antagonist, insomnia, anxiety, addiction, neuroscience

The current special topics issue of Frontiers in Neuroscience “Insomnia and Beyond—Exploring the Therapeutic Potential of Orexin Receptor Antagonists” comprises 20 papers from leaders in the orexin/hypocretin field presenting their latest findings and thorough reviews, and will undoubtedly serve as an important reference for others engaged in this field of study. Since the independent identification of the orexin/hypocretin system in 1998 by two teams investigating hypothalamic signaling (de Lecea et al., 1998; Sakurai et al., 1998), the field has rapidly expanded to include potential roles of orexin in processes as diverse as sleep/wake, addiction, feeding, pain and anxiety. Orexin neuropeptides (OX-A, OX-B) are cleaved from a common precursor and secreted from orexinergic neurons localized in the lateral hypothalamus. Orexins (also passionately referred to as hypocretins by many investigators) bind and activate two G-protein coupled receptors (OX_1_R and OX_2_R), with OX-A binding equally, and OX-B having ~10 fold selectivity for OX_2_R. Although orexinergic neurons are localized to less than 100,000 hypothalamic neurons in the human brain, orexin receptors exhibit overlapping and distinct expression throughout the CNS, with significant expression in brain regions associated with arousal and vigilance state, as well as reward and stress processing.

Following their seminal discovery of this novel neuropeptide system 16 years ago, authors of the foundational *Proc. Natl. Acad. Sci*. and *Cell* papers joined by other exceptional researchers provide their latest insights into the function of orexin signaling and its therapeutic potential.

Drs. de Lecea and Huerta (2014) explain their current view on the interaction of orexin neurons with other neuromodulatory systems based on recent optogenetic experiments. A paper from Kohlmeier et al. (2013) presents an original investigation on the interaction of the orexin system with cholinergic and monoaminergic neurons using OX_1_ and OX_2_ receptor knockout mice. Two separate papers from Etori et al. (2014) and Tortorella et al. (2013) provide insight into potential synaptic interactions between the hypothalamus, locus coeruleus and oral pontine reticular nucleus, suggesting that orexin signaling within this circuit modulates stage succession during sleep-wakefulness cycles. Expanding upon the first observations of genetic disruptions of orexin receptors in dogs, Thompson et al. (2014) provide a pharmacogenetic overview of orexin peptides and their receptors, and describe the potential impact of polymorphisms observed in human genetic studies. Finally, work described in Flores et al. (2013) illustrates cannabinoid-hypocretin cross-talk within the CNS, an emerging area of interest for the field.

The identification of a neuropeptide system with restricted expression, key functions in fundamental CNS processes and approachability of discrete GPCR subtypes has made orexin receptors valuable targets for the next generation of CNS therapeutics, with multiple teams actively characterizing small molecules to understand the pharmacology and potential therapeutic applications of targeting the orexin system. Equihua et al. (2013) takes the reader on a journey comparing the merits and challenges of orexin receptor antagonists as potential sleep medications relative to currently used hypnotics. A paper from Callander et al. (2013) representing a team formerly at Novartis, provides substantial *in vitro* characterization and describes differential kinetic properties of dual orexin receptor antagonists (DORAs) that have undergone clinical evaluation. Two pharmacological papers from Morairty et al. (2014) (work by groups UCSF and SRI), and Ramirez et al. (2013) (Merck Research Laboratories), demonstrate that DORAs lack the impairment on motor and cognitive function in rodents exhibited by positive GABA-A receptor modulators. Etori et al. (2014) (Kanazawa University), Dugovic et al. (2014) (Janssen), and Hoyer et al. (2013) (team formerly at Novartis) explore the potential differential impact of DORAs as well as antagonists selective for OX_1_R and OX_2_R on non-REM and REM sleep architecture.

With regard to other potential indications for OXR antagonists beyond insomnia, two manuscripts from teams at Lilly Research Laboratories and MUSC (Anderson et al., 2014; Fitch et al., 2014) describe a novel OX_2_R selective antagonist, and its effects on rodent models of ethanol addiction and depression relative to a DORA and OX_1_R selective antagonist. In addition, (Steiner et al., 2013) (Actelion), investigated the impact of chronic pharmacological OX_1_R blockade in a rat model of diet-induced obesity.

A further area of orexin research which is being actively investigated focuses on the impact of orexin signaling on sympathetic autonomous nervous system activation, both during sleep and wake stages, as well as during heightened arousal and stress. Two excellent reviews from leaders in the field (Carrive, 2013; Li and Nattie, 2014) provide an overview of the current perspectives regarding cardiovascular and respiratory control by orexins. (Colas et al., 2014) present evidence supporting the existence of an orexin circuit between central and peripheral nervous system, where orexin signaling potentially modulates sensory processing at dorsal root ganglia.

Together with the increasing availability of pharmacological tools, investigations of the role of orexin in limbic emotional processing have also intensified during the last 3–4 years. Two detailed reviews shed light on this topic, from both academic and industry perspectives (Yeoh et al., 2014 from the University of Newcastle and the Hunter Medical Research Institute; Merlo Pich and Melotto, 2014, formerly of Glaxo Smith Kline). These two papers outline recent findings from rodent studies identifying involvement of orexins in mediating compulsive behavior, as well as panic, depression and anxiety-like reactions during withdrawal from drugs of abuse.

Importantly, this special issue highlights the exquisite foundational and translational work being conducted by teams at leading academic and biopharmaceutical laboratories, focused on investigating a range of therapeutic opportunities. Although it has only been 16 years since the identification of the orexin/hypocretin system, the wealth of knowledge obtained thus far has set the stage for the next decade of orexin research. It is with great appreciation to the contributing authors and with much enthusiasm that we present this special topics issue of Frontiers in Neuroscience.

## Conflict of interest statement

Michel Steiner is employed by Actelion Pharmaceuticals. Christopher Winrow is employed by Merck. Both companies are actively involved in the development of orexin receptor antagonists for clinical applications.
